# Targeting delivery and minimizing epidermal diffusion of tranexamic acid by hyaluronic acid-coated liposome nanogels for topical hyperpigmentation treatment

**DOI:** 10.1080/10717544.2021.1983081

**Published:** 2021-10-01

**Authors:** Ying Liu, Yue Han, Tingting Zhu, Xianglei Wu, Wenxin Yu, Jiafang Zhu, Ying Shang, Xiaoxi Lin, Tianlan Zhao

**Affiliations:** a Department of Plastic and Cosmetic Surgery, The Second Affiliated Hospital of Soochow University, Suzhou, China; b Department of Laser and Aesthetic Medicine, Department of Plastic and Reconstructive Surgery, Shanghai Ninth People's Hospital, Shanghai Jiao Tong University School of Medicine, Shanghai, China; c Department of Dermatology, The First Affiliated Hospital of Soochow University, Suzhou, China

**Keywords:** Liposome nanogels, minimizing epidermal diffusion, targeting delivery, tranexamic acid, topical hyperpigmentation treatment

## Abstract

Hyperpigmentation is a common complaint and distressing problem in dermatology, and tranexamic acid (TA) is an effective treatment agent but limited by the delivery to melanocytes in the epidermis. Herein, a novel TA naogels (named HA/TA-LP), combining the advantages of liposomes and hyaluronic acid (HA), are prepared and assessed for topical hyperpigmentation treatment with targeting delivery and minimizing epidermal diffusion. Morphological characteristics indicate numerous TA-loaded liposomes packed in HA gels. *In vitr*o cell studies using human A375 melanoma cells show that HA/TA-LP can promote the uptake of TA by targeting delivery with resulting inhibition of tyrosinase activity and melanin production. Guinea pigs are used to construct hyperpigmentation models and investigate the topical delivery and treatment efficacy of HA/TA-LP. *In vivo* topical delivery studies indicate HA/TA-LP realize the effective delivery into melanocytes with an ideal balance of effective permeability and minimizing epidermal diffusion. Subsequently, hyperpigmentation treatment assessments reveal that HA/TA-LP inhibit tyrosinase activity and melanin production under the radiation of UVB. Our study identifies favorable properties of HA/TA-LP for treating hyperpigmentation, and provides an experimental basis for further clinical application.

## 1. Introduction

Hyperpigmentation is a common complaint and distressing problem in dermatology practice (Hu et al., 2021; Say et al., 2021). Clinically, it is classified into diffuse, circumscribed, linear, or reticulated variants. Notably, hyperpigmentation of skin can almost immediately manifest through causes including inflammation (caused by acne, eczema, and contact dermatitis), cutaneous injury and UVB irradiation (Alkhowailed et al., 2021). Its pathogenesis results from melanocytes being exposed to paracrine melanogenic factors which can upregulate melanogenesis-associated enzymes (such as tyrosinase), resulting in the production of melanosomes (Peters, 2005). Biochemically, tyrosinase is related to melanosome production, and serves as the major target of hyperpigmentation treatment (Ohbayashi and Fukuda, 2020). Therefore, reducing melanosomes by inhibiting tyrosinase activity has become a primary goal of hyperpigmentation treatment. Among various tyrosinase activity inhibitors, tranexamic acid (TA), an anti-fibrinolytic drug, can inhibit tyrosinase activity in melanocytes by suppressing prostaglandin synthesis and epidermal plasmin activity (Cohen, 2018; Rohrich & Cho, 2018; Vogel et al., 2018). Indeed, TA is a widely used skin-lightening agent in clinically treating melasma (Taylor et al., 2006; Ho et al., 2011; Shenoy & Madan, 2020). Considering its higher efficiency in reducing pigmentation compared to vitamin C or glycolic acid, TA is also an ideal candidate for hyperpigmentation treatment (da Silva Souza et al., 2021; Tawfic et al., 2021).

The delivery of TA into melanocytes becomes the challenge of hyperpigmentation treatment (Mobasher et al., 2020; Nautiyal & Wairkar, 2021). Although the intradermal injection and oral administration are used in clinical application, they are each problematic, the former being invasive while the latter suffers from efficacy issues (Bala et al., 2018; Wang et al., 2019). In contrast, topical delivery is an attractive alternative in dermatology, improving drug utilization as well as eliminating potential systemic side effects (Sabir et al., 2019; Batool et al., 2021). However, TA, a hydrophilic molecules, is inefficient for topical delivery into melanocytes in the basal layer of epidermis for following reasons: (1) TA is difficult to pass through lipid barriers of the stratum corneum (SC); (2) TA is poorly retained in basal layer of epidermis to adequately enter melanocytes (Kim et al., 2016). Therefore, the effective delivery of TA into melanocytes is the primary concern in hyperpigmentation treatment. In this regard, topical formulations of TA must be able to overcome the SC barrier and be sufficiently retained in the epidermis to target melanocytes.

Recently, nanotechnologies have proved to be helpful tools in preparing TA formulations for hyperpigmentation treatment (Din et al., 2017; Xing et al., 2021). Here liposomes represent a novel lipid nano-delivery system, exhibiting high efficacy in topical drug delivery (Oh et al., 2016; Wang et al., 2018). On this basis, TA-loaded liposomes (TA-LP) potentially represent an efficient formulation for topical delivery of TA. However, TA-LP has fallen short of expectations, because of against the epidermal retention for spurious penetrability and lacking targeting ability. Therefore, improving the effective delivery of TA-LP requires modifications that would target melanocytes and minimize diffusion in the epidermis with characteristics of stability and adhesion. In this regard, hyaluronic acid (HA) presents several advantages: (1) HA can loosen corneocyte packing to overcome SC barriers by its hydrophobic patch domain (Brown and Jones, 2005; Yang et al., 2012); (2) HA can actively adhere to melanocytes utilizing cell surface HA receptors (such as CD44), thereby promoting the targeted delivery to melanocytes (Ahrens et al., 2001); (3) HA chains can pack the TA-LP as the viscous nanogels by the electrostatic interaction between HA chains and phospholipid molecules, which limits the penetrability of TA-LP for the minimizing epidermal diffusion (Chen et al., 2020). Therefore, how to prepare HA modified TA-LP with features of targeting delivery and minimizing epidermal diffusion is essential to hyperpigmentation treatment.

**Scheme 1.**
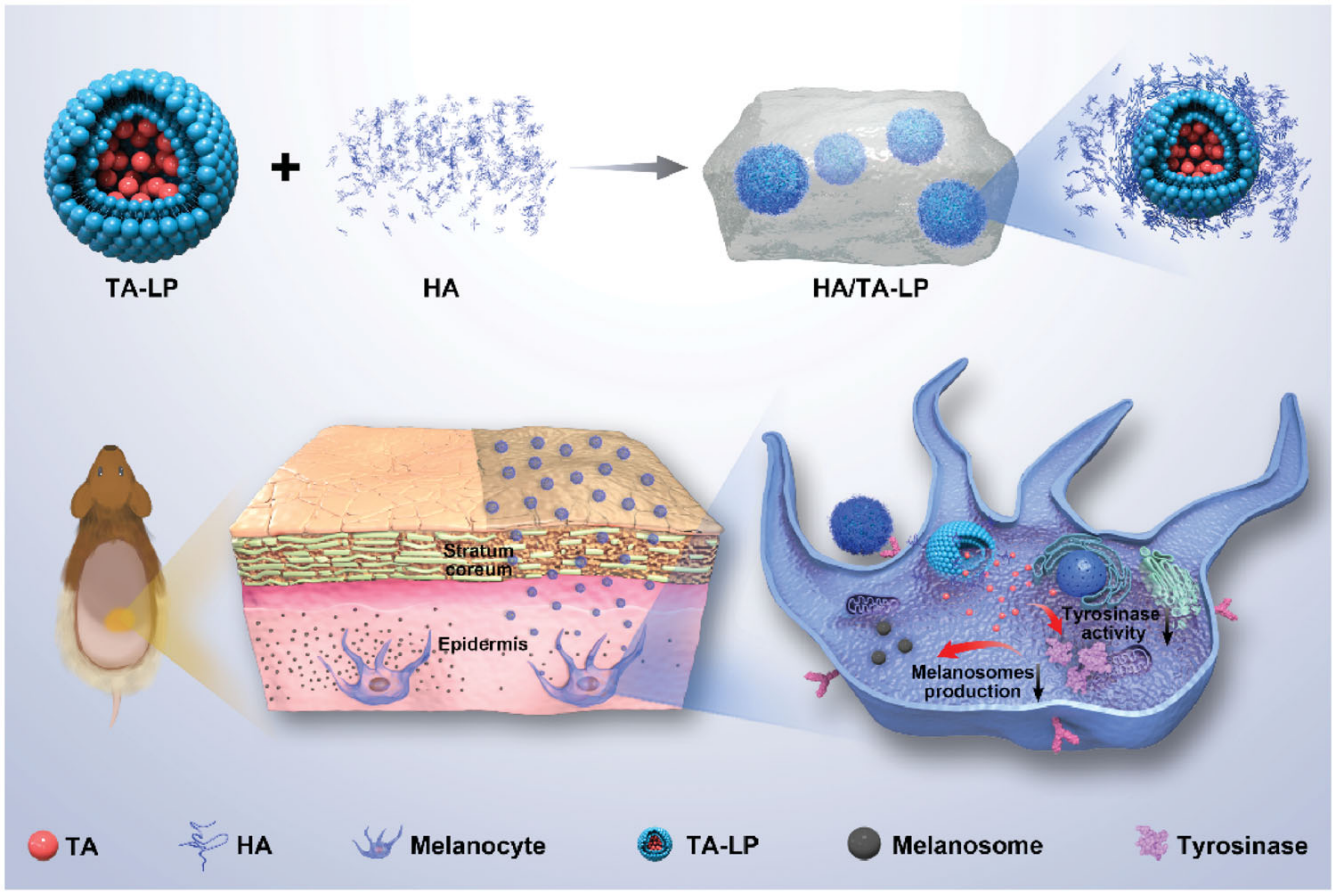
Schematic illustration of the fabrication and application of HA/TA-LP.

Herein, topical TA naogels (named HA/TA-LP), combining the advantages of liposome and HA, were prepared for hyperpigmentation treatment with targeting delivery and minimizing epidermal diffusion. First, *in vitro* cell studies using human melanoma cells showed that HA/TA-LP promoted the uptake of TA, resulting in the inhibition of tyrosinase activity and melanin production. Secondly, *in vivo* the topical delivery and treatment efficacy were investigated using guinea pigs as hyperpigmentation models, and the results indicated that HA/TA-LP could effectively retain in epidermis, enter melanocytes and inhibit tyrosinase activity and melanin production under UVB radiation. Thus, our studies suggested that HA/TA-LP was the ideal TA nanogels for topical hyperpigmentation treatment with targeting delivery and minimizing epidermal diffusion (as shown in Scheme 1). Moreover, such findings provided the experimental basis for the application of HA/TA-LP in clinical practice.

## 2. Experimental section

### 2.1. Preparation and characterization of HA/TA-LP

TA-LP were prepared using the ethanol injection method with some modifications at first (Sebaaly et al., 2016; Li et al., 2021). Briefly, soy phosphatidylcholine (SPC, AVT, Shanghai, China) and TA (Aladdin, Shanghai, China) were first dissolved in 1 mL ethanol before dropwise addition into 5 mL deionized H_2_O to form a milky aqueous phase. TA-LP (SPC: 10 mg/mL and TA: 5 mg/mL) were obtained by removing the superfluous ethanol using evaporation under a reduced pressure using a vacuum oven. Finally, HA (50 mg, 100 KDa; Aladdin, Shanghai) was added to the TA-LP dispersion, and the mixture stirred overnight to hydrate as the final HA/TA-LP preparation. Where indicated, the fluorescent dye Rhodamine 6 G (R6G, 0.1 mM) replaced TA in the nanogels using the same preparation procedure. A control HA-TA gels were consisted of a mixture of HA (50 mg) and TA solution (5 mg/mL, 5 mL).

TA-LP and ultrathin HA/TA-LP gel samples (100 nm thickness, cryosectioned by cryoul-tramicrotome,UC6/FC6, Leica Austria) were examined by transmission electron microscopy (TEM; Tecnai G2, FEI, CA, USA) and scanning electron microscopy (SEM; JSM-6360LA, JEOL, Tokyo, Japan). *In vitro* TA release was evaluated according to the dialysis method (Zhang et al., 2016). TA was derivatized with *o*-phthalaldehyde (the most sensitive fluorogenic compounds available for reaction with TA) and detected by high-performance liquid chromatography (Hsieh et al., 2013; Silva et al., 2017). Size distribution was determined by dynamic light scattering (DLS; NiComp 380ZLS, Santa Barbara, USA) at 25 °C.

### 2.2. Cell culture and animal model construction

Human melanoma cells (A375) were used as melanocytes for *in vitro* studies. The cells were grown in Dulbecco's-modified Eagle's medium (DMEM; HyClone, Logan, Utah, USA) containing 10% fetal bovine serum (FBS; Hyclone, Thermo Scientific, Epsom, UK) at 37 °C with 5% CO_2._

For the *in vivo* studies, hyperpigmentation was induced on the backs of brown guinea pigs (the hair had been shaved) by exposure to UVB radiation three times a week for four weeks (500 mJ/cm^2,^ Waldmann UV 800, emitting 280–305 nm). Nanogels were topically applied to pigmentation areas every two days for two weeks. Before skin tissue collection, guinea pigs were painlessly sacrificed by overdose of pentobarbital sodium. Animals were maintained under constant conditions with all procedures performed under the approval of the Animal Experimentation Ethics Committee of Soochow University.

### 2.3. Cytotoxicity and cell uptake studies

To assess cell cytotoxicity, A375 cells were seeded in 96-well plates and cultured for 24 h. After replaced with fresh medium containing a concentration series of HA/TA-LP or HA-TA, A375 was incubated for another 24 h, and then cell viabilities were evaluated using MTT assay kit (Trevigen, Gaithersburg, MD, USA) following the manufacturer’s instructions.

After a further 24 h, cell viabilities were evaluated using the MTT assay following the manufacturer’s instructions. For the cellular uptake studies, A375 were seeded in 35 mm glass bottom plates and cultured for 24 h. Next, rhodamine 6 G (R6G; Aladdin, Shanghai, China)-replaced HA/TA-LP was incubated with the cells for 2 and 6 h, respectively. Then, the cells were detected using confocal laser scanning microscope (CLSM, Leica TCS SP8; Leica Microsystems GmbH, Germany) with 488 nm excitation and 650 nm emission for R6G. Additionally, A375 treated with HA/TA-LP were collected and carried out standard TEM sample preparation methods for TEM observation.

### 2.4. Assessment of tyrosinase activity and melanin content

Tyrosinase activity was measured using a spectrophotometric method (Noh et al., 2020). A375cells were grown in 24-well plates and treated with HA/TA-LP and HA-TA or Control for 24 h. The collected cells were disrupted by sonication and centrifugation, and then quantified using a protein assay kit (Bio‐Rad, Richmond, CA, USA). The reaction mixtures were incubated in a 96-well plate for and their absorbance was measured at 450 nm using a Synergy H4 hybrid reader (Bio-Tek, VT, USA). The melanin content was determined according to the reported procedure (Teng et al., 2020). A375 were seeded into a six-well plate for 24 h, and then treated with HA/TA-LP and HA-TA for 24 h. The collected cells were re-suspended in 1 mL NaOH (1 M, containing 10% DMSO, *v/v*) and heated at 80 °C for 1 h. The absorbance (405 nm) of solution was read. The data were expressed as the percentage of the blank control.

### 2.5. *In vivo* topical delivery

R6G-replaced nanogels were topically applied to pigmentation areas and skin tissues collected after 1, 2, and 6 h. For the observation of TA distribution, skin tissues were embedded in O.C.T compound, cut into sections (10 μm-thick, perpendicular to the surface), and affixed to polysine-coated glass slides and then incubated with 4′, 6-diamidino-2-phenylindole (DAPI) staining solution. Then, the samples were detected using CLSM. Furthermore, skin tissues collected at 2 and 6 h were processed using standard methods for TEM.

### 2.6. Assessment of hyperpigmentation treatment

12 guinea pigs were divided to two groups (HA/TA-LP and TA-HA), and each guinea pig contained the treating area (applied with formation) and Control area (no any operation). Nanogels were topically applied to the pigmentation areas every two days for two weeks with visual appearance used to gauge the effects of the treatments. Furthermore, Antera 3 D with melanin mode (Miravex, Dublin, Ireland) was used to map the distribution of melanin and measure its average concentration and uniformity in the pigmented areas. Afterwards, skin tissues were harvested for histological analysis using hematoxylin and eosin (HE) stain with melanocytes visualized with Fontana–Masson (F-M) staining against melanin. F-M positive cells were counted in a 500 × 400 mm area in 10 different fields (Ookubo et al., 2014). Furthermore, the skin tissue pieces (1 × 1 × 1 mm) were prepared for TEM examination using standard methods.

### 2.7. Statistical analysis

Data are presented as the average ± SD. Statistical significance was determined employing analysis of variance (ANOVA) using SPSS 18.0 software (SPSS, IL, USA) with *p*<.05 as a minimal level of significance.

## 3. Results and discussion

### 3.1. Characterization studies

**Figure 1.**
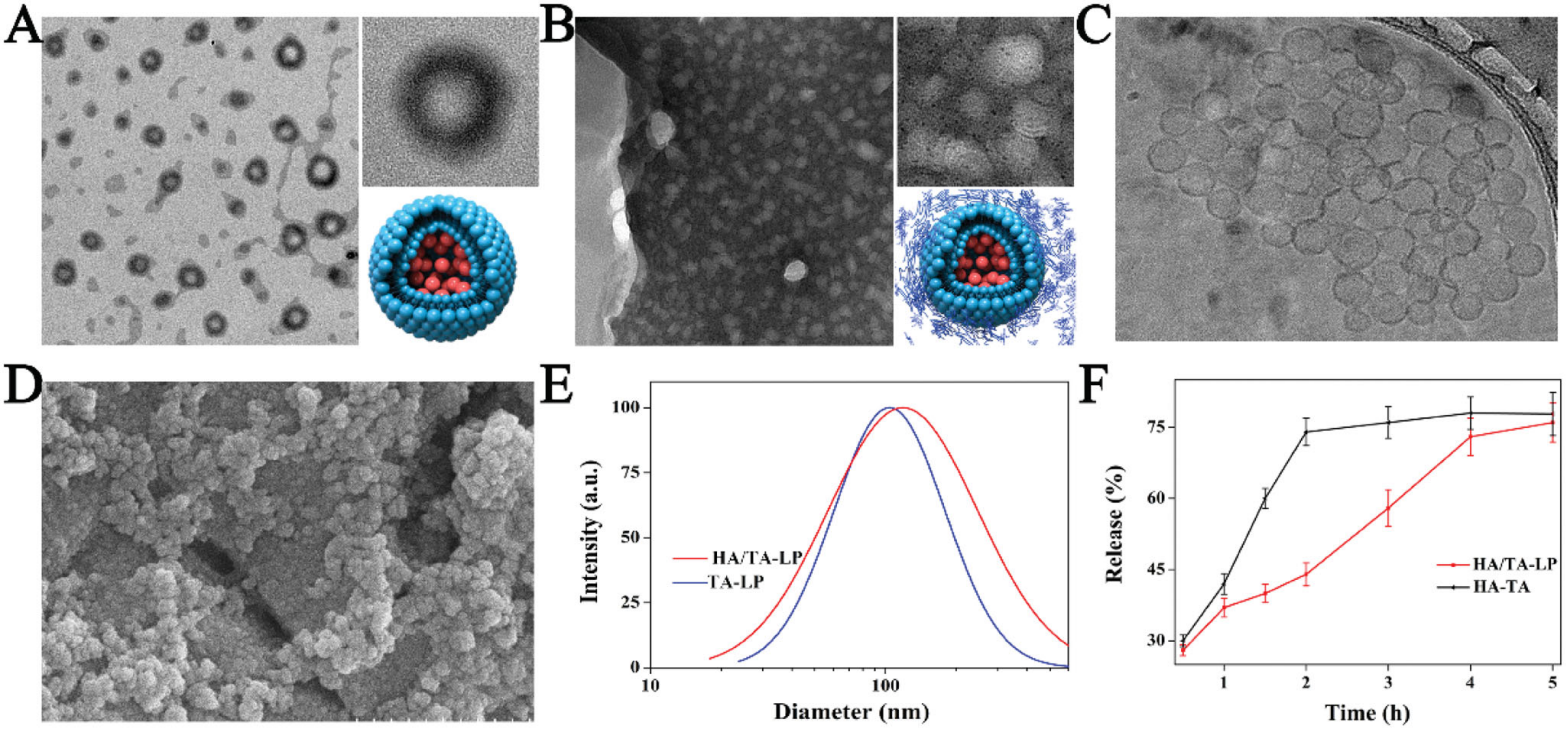
(A, B) TEM and schematic images of TA-LP and HA/TA-LP nanogels, (C, D) Cryo-TEM and SEM images of HA/TA-LP, and (E, F) the diameter distributions and TA release profiles of HA/TA-LP and HA-TA.

The morphological features of TA-LP and HA/TA-LP were first examined using TEM. TA-LP were generally spherical in shape with an internal aqueous core (Figure 1(A)). HA/TA-LP were cryosectioned to reveal TA-LP distribution, indicating that abundant TA-LP homogenously dispersed in the gel matrix (Figure 1(B)). The similar structure of HA/TA-LP was also verified by Cryo-TEM (Figure 1(C)). It was clear that abundant TA-LP homogenously distributing in gel matrix. Meanwhile, SEM image also indicted that TA-LP was densely distributed in gel matrix according to a topography effect (Figure 1(D)). Compared with TA-LP, HA/TA-LP exhibited a slightly increased size (122 ± 34 nm to 104 ± 42 nm), likely because HA chains were adsorbed on the surface of TA-LP by electrostatic interactions between SPC and HA chains (Figure 1(E)). In addition, TA release was also studied (Figure 1(F)). HA-TA produced a fast TA release in 2 h. In contrast, HA/TA-LP exhibited a different release pattern. TA loaded by HA was released in 1 h, a slower release indicated entrapped TA barely leaked out in following 1 h because TA-LP was still stable. Subsequently a burst release due to TA released from ruptured TA-LP. Therefore, the release profile suggested that HA/TA-LP could realize a stable delivery and a fast release in its application. Furthermore, the encapsulation efficiency of TA was calculated as TA releasing from ruptured TA-LP, and it was satisfactory (33.1% ± 5.2%).

Sum to up, HA/TA-LP nanogels had the unique structure of abundant TA-LP were packed by HA chains, which possessed the ability of topical and targeting ability.

### 3.2. Cellular viability and uptake studies

**Figure 2.**
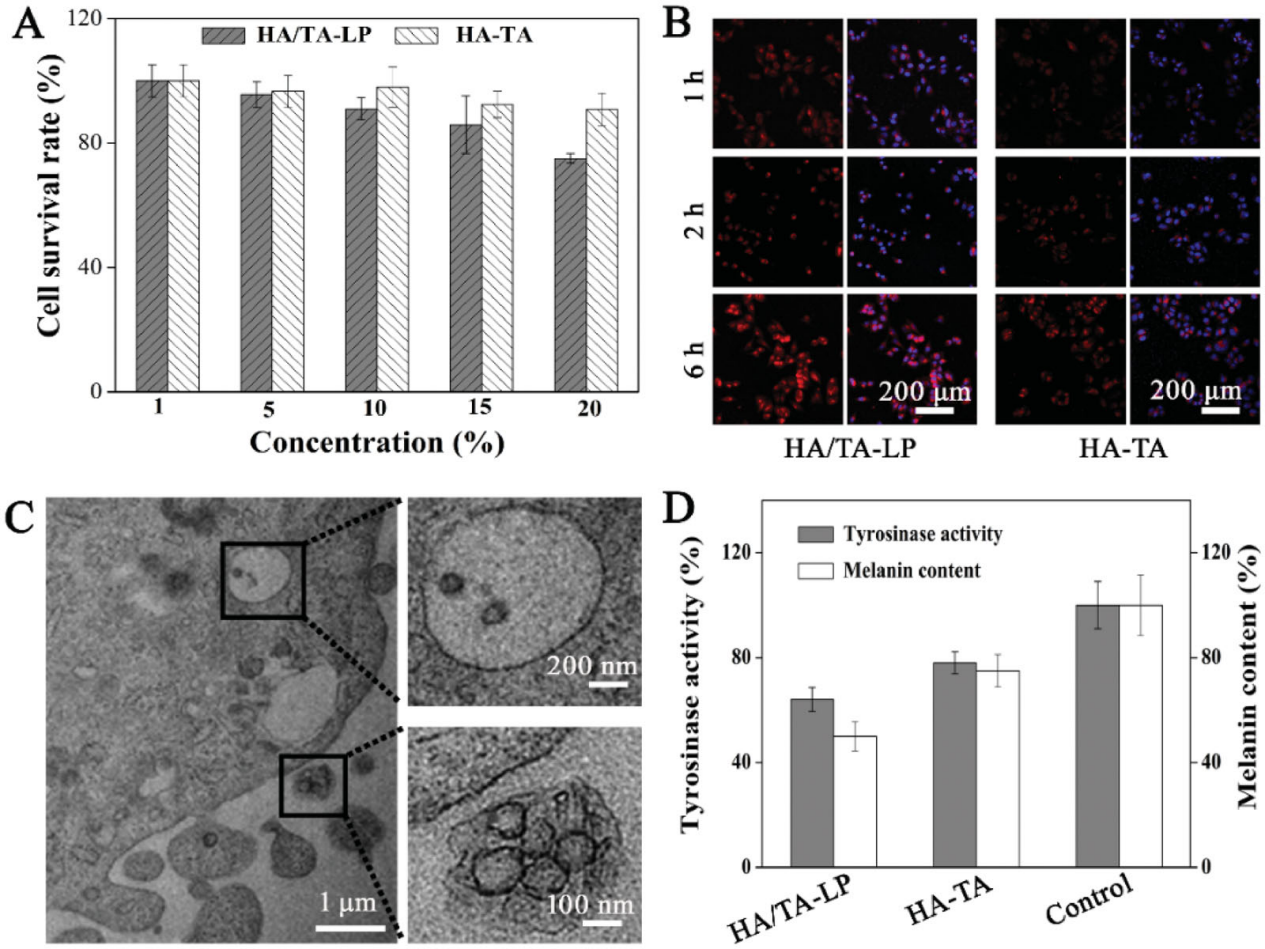
(A) The cell viability of A375 cells treated with HA/TA-LP or HA-TA; (B) CLSM images of uptake of TA by A375 cells (red, indicated by R6G); (C) Ultrastructural characterizations of cell treated with HA/TA-LP; (D) Effects of HA/TA-LP and HA-TA on tyrosinase activity and melanin content (*p* < .05).

The cytocompatibility of HA/TA-LP was also a crucial indicator for potential applications, which was evaluated using MTT (Figure 2(A)). The results of these assays revealed there was no significant cytotoxicity at concentrations of HA/TA-LP <10% (cellular viability >90%). Concentrations of 15 and 20% HA/TA-LP produced significantly lower viability (<85%). HA-TA at all concentrations did not affect the cellular viability (>90%). On this basis 10% HA/TA-LP (0.5 mg/mL TA) was used in downstream cell studies.

First, the cellular uptake of HA/TA-LP using R6G instead of TA (red fluorescence). Examination of the fluorescence profiles of cells indicated their intensity gradually increased with incubation time, indicating that TA was delivered into cells (Figure 2(B)). The fluorescence intensities further indicated that HA/TA-LP were better in promoting the uptake of TA. Furthermore, the targeting delivery of HA/TA-LP was further investigated using TEM (Figure 2(C)). HA/TA-LP containing numerous TA-LP aggregated at the cell surface. Moreover, TA-LP was evident in the cytoplasm taken within pinocytotic vesicles, indicating TA-LP enters cells through a pinocytotic-like mechanism. Taken together, these experiments indicated that HA improved the targeted cellular delivery of TA.

### 3.3. Cellular tyrosinase activity and melanin content

We then sought to confirm whether HA/TA-LP resulted in decreased measures of pigmentation was sought to confirm in following. Toward this, A375 cells were treated with HA/TA-LP or HA-TA before measuring cellular tyrosinase activity and melanin content (Figure 2(D)). As expected, HA-TA inhibited tyrosinase activity compared with the control group. However, HA/TA-LP resulted in higher inhibition efficiency compared to HA-TA. Analysis of cellular melanin content showed the same trend where HA/TA-LP reduced melanin content more significantly than HA-TA. Compared with the reported works, HA/TA-LP exhibited the better performance in inhibiting tyrosinase activity and reducing melanin content, because of the better delivery efficiency to cells (Hsieh et al., 2013).

Sum up, the results establish that HA/TA-LP exhibits excellent cytocompatibility, with the inclusion of HA providing improving the targeted delivery and cellular uptake of TA. In turn, the increased uptake of TA resulted in improved inhibition of tyrosinase activity and reduction in melanin content.

### 3.4. *In vivo* topical delivery

**Figure 3.**
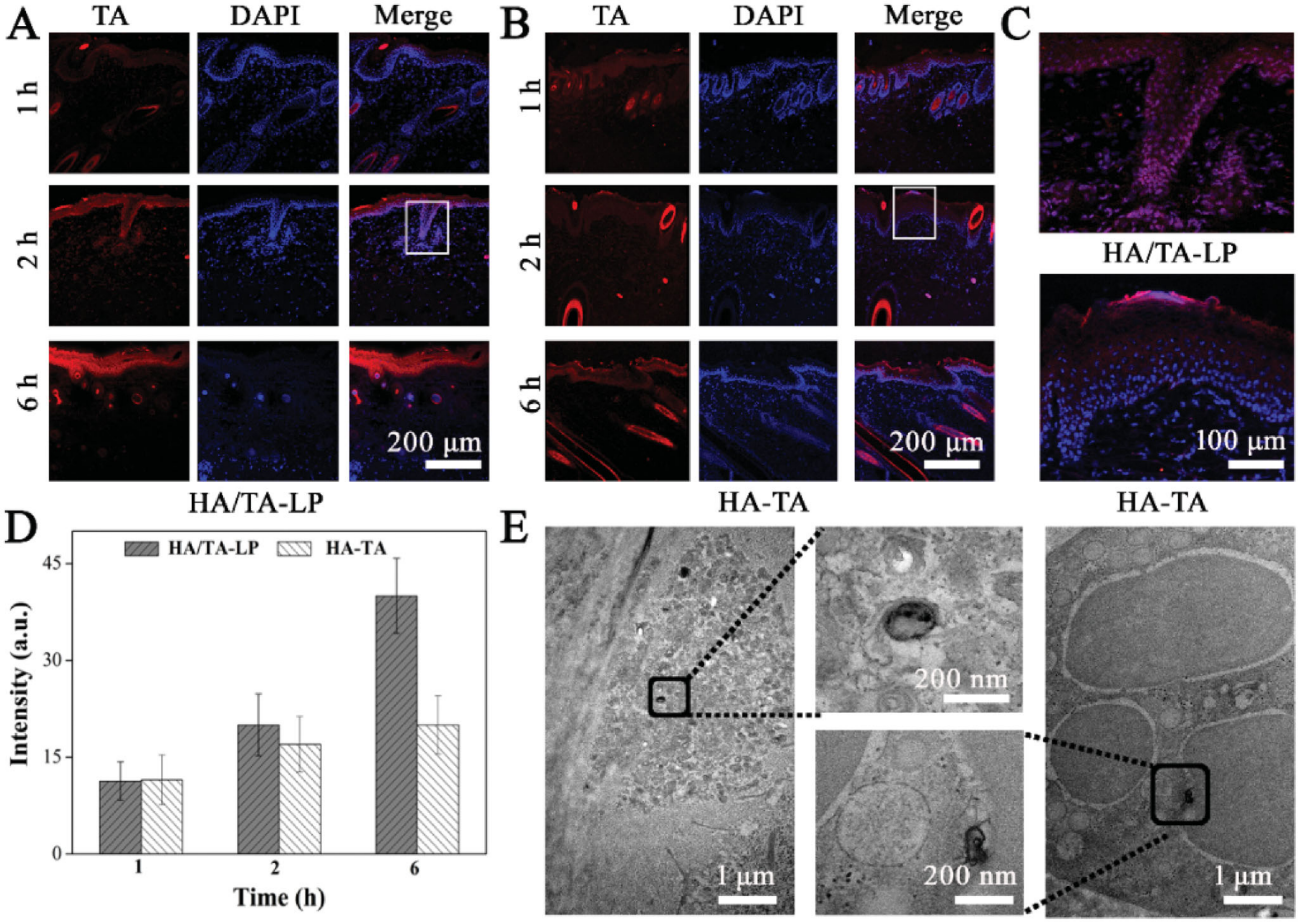
(A, B) Fluorescence images of skin tissue after *in vivo* administration with HA/TA-LP and HA-TA for different formulations (TA: red fluorescence, indicted by R6G); (C) The details in white frames; (D) Statistics of mean red fluorescence intensity; (E) TEM image of HA/TA-LP *in vivo* delivering in epidermis and into melanocytes.

That HA/TA-LP nanogels topically penetrated into epidermis with minimizing diffusion and targeted delivered TA into melanocytes was the premise in TA treating hyperpigmentation. Therefore, HA/TA-LP was topically applied in guinea pig models, and the *in vivo* distribution and accumulation of TA (inducted using R6G) were visually investigated (Figure 3(A,B)). HA could loosen corneocyte packing to overcome SC barriers, and consequently, TA easily crossed the SC and fluorescence was mainly distributed in epidermis in both HA/TA-LP and HA-TA groups, suggesting that diffusion was minimized within the epidermis. For HA-TA, there was no obvious fluorescence increases at different administration times. On contrast, HA/TA-LP produced much stronger fluorescence, indicating it promoted much more TA penetrate into epidermis. Interestingly, compared with HA-TA, HA/TA-LP group had many fluorescence intense spots in epidermis at 2 h. After amplifying, the co-localization of red and blue fluorescence suggested that HA/TA-LP had effectively entered and accumulated in melanocytes (Figure 3(C)). The statistics of fluorescence intensities confirmed that HA/TA-LP could enhanced 2-folds TA retention in epidermis (Figure 3(D)). Furthermore, the ultrastructural characterizations of topical delivery were also verified using TEM (Figure 3(E)). HA/TA-LP had passed through SC and approached the surface of cell in epidermis, indicating HA/TA-LP had the target ability to melanocytes. Notably, HA/TA-LP remained present in the aggregated gel structure, which would likely help to limit its diffusion. Furthermore, intact TA-LP was observed within melanocytes which could be identified by their numerous melanosomes, providing clear evidence that HA/TA-LP successfully delivered TA into the correct target cells. Therefore, the results of the topical delivery studies indicated that HA/TA-LP could penetrate into epidermis with minimizing diffusion, and then targeted delivered TA into melanocytes.

### 3.5. *In vivo* hyperpigmentation treatment

**Figure 4.**
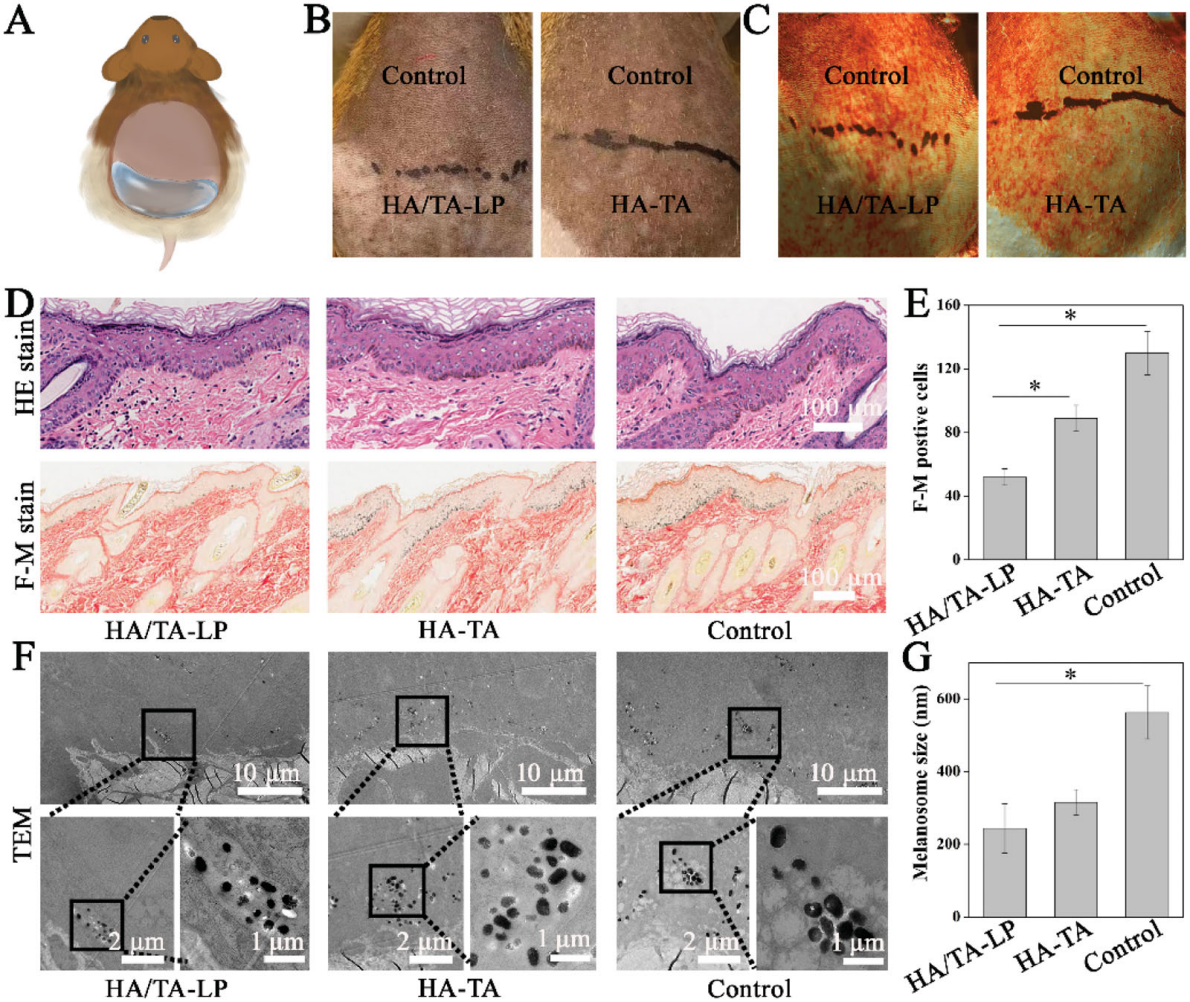
**(**A) Schematic of topical hyperpigmentation treatment; (B, C) pigmentation alterations and melanin distributions in the different groups; (D) HE staining and F-M staining images; (E) statistical analysis of F-M positive cells (*: *p* < .05); (F) Ultrastructural characterizations of skin tissues in in the different groups; (G) statistical analysis of melanosomes size (*: *p* < .05).

HA/TA-LP was topically applied in pigmentation areas as schematic presentation (Figure 4(A)). With UVB irradiation, the melanin of guinea pig skin was continually deposited, and treatment efficiency was evaluated by appearance observation and histopathological analysis. After treatment, there were differential pigmentation alterations and melanin distributions in the different groups (Figure 4(B,C)). Compared with the HA-TA group, the HA/TA-LP group exhibited visibly decreased hyperpigmentation levels. Histopathological analysis of the skin sections using HE staining and F-M staining confirmed that the HA/TA-LP group had much less melanin deposition in the basal layer of the epidermis relative to the HA-TA and control groups (Figure 4(D)). Moreover, specific F-M staining for melanocytes showed that HA/TA-LP treatment significantly reduced melanin production, represented by the F-M positive cells compared with other groups (Figure 4(E)).

The distribution and content of melanosomes were important features for hyperpigmentation, which was visual to evaluate the hyperpigmentation treatment using TEM (Figure 4(F)). A larger number of melanosomes were present in the epidermis of the control group, which appeared similar to the HA-TA group samples. In contrast, much less melanosomes were evident in the epidermis in the HA/TA-LP group. Furthermore, the aggregated melanosomes were present within the basal layer, indicating that they existed in the same melanocytes. Compared with other treatment groups, there were fewer melanosomes in melanocytes in the HA/TA-LP group, indicating that HA/TA-LP inhibited melanosome production. Further image analysis also revealed that HA/TA-LP significantly inhibited melanosome production through decreases in melanosome size (Figure 4(G)). Therefore, both histopathological analysis and TEM observations clearly suggest that HA/TA-LP could inhibit tyrosinase activity and melanin production following UVB radiation, exhibiting high efficiency in hyperpigmentation treatment after topical application. Compared to reported microneedle delivering TA, HA/TA-LP presented the better performance because it could effectively deliver TA to target cells, because its satisfactory EE and properties of targeting delivery and minimizing epidermal diffusion (Zhang et al., 2020).

## 4. Conclusions

To our best knowledge, this study represents the first report investigating topical TA nanogels (named HA/TA-LP) for hyperpigmentation treatment. According to the results of *in vitro* cell studies, HA/TA-LP promoted the uptake of TA by its targeting delivery, inhibited the tyrosinase activity and reduced the melanin content. The *in vivo* topical delivery studies indicated HA/TA-LP had the ideal balance of effective permeability and minimizing epidermal diffusion. Subsequently, *in vivo* treatment assessments revealed that HA/TA-LP could be topically delivered into melanocytes to inhibit tyrosinase activity and melanin production under the radiation of UVB. Thus, our study provides a firm experimental basis for the further development of HA/TA-LP in clinical applications.
